# The role of childhood maltreatment, current stress, and alexithymia in nonsuicidal self-injury: A sex-specific analysis among Korean young adults

**DOI:** 10.1371/journal.pone.0340384

**Published:** 2026-04-06

**Authors:** Eunjin Jo, Gyumyoung Kim, Ji-Won Hur

**Affiliations:** School of Psychology, Korea University, Seongbuk-gu, Seoul, Korea; Tehran University of Medical Sciences, IRAN, ISLAMIC REPUBLIC OF

## Abstract

Prior research has highlighted various risk factors for nonsuicidal self-injury (NSSI), yet inconsistencies remain regarding their predictive value and sex-specific effects. This study examined how childhood maltreatment, current perceived stress, and alexithymia contribute to the engagement and severity of NSSI, with a focus on sex differences. A total of 731 individuals participated in this study (233 males, 498 females). The sample consisted of 481 individuals with NSSI (124 males, 357 females) and 250 control participants without a history of NSSI (109 males, 141 females). Logistic and multiple regression analyses were conducted separately by sex to test the differential predictive value of the factors. Among males, physical abuse significantly predicted NSSI engagement, and current stress was uniquely linked to greater NSSI method versatility. Conversely, in females, emotional abuse, current stress, and alexithymia emerged as key predictors of NSSI engagement, while sexual abuse, physical neglect, and alexithymia predicted greater severity. These findings underscore the existence of distinct, sex-specific pathways linking early adversity, emotional processing difficulties, and proximal stress to both the engagement and severity of NSSI. Tailoring prevention and intervention strategies to account for these sex-specific patterns is required to improve clinical outcomes.

## Introduction

In recent years, considerable attention has been directed towards understanding the complex interplay between sex and nonsuicidal self-injury (NSSI), a behavior characterized by deliberate, direct damage to bodily tissue without suicidal intent. A meta-analysis estimated the prevalence of NSSI to be 13.4% among young adults [1]. More recently, a multinational study of first-year college students reported a lifetime prevalence as high as 17.7%, with engagement in NSSI associated with an increased risk of the onset of mental disorder [2]. Furthermore, given that another meta-analysis [3] has suggested that NSSI behaviors paradoxically increase suicide risk, there is no doubt that NSSI has been recognized as a crucial, worldwide public health concern. Several attempts have been made to identify factors contributing to NSSI to develop appropriate intervention strategies, which encompass various approaches, including psychological [4] and pharmacological interventions [5] aimed at mitigating associated clinical risks, such as suicidal behavior.

Despite the clear global health imperative, the role of sex in NSSI risk remains inconsistent. While the female gender has frequently been identified as a potential risk factor for NSSI globally [6–8], this pattern is inconsistent across regions. Specifically, meta-analytic findings published around the same period have presented that sex differences in NSSI prevalence were less pronounced among Asian youth populations compared to those in other continents [7,8]. Globally, NSSI is significantly more prevalent among females, yet in Asian adolescents, this sex difference is attenuated [8] or absent [7]. This unique regional deviation, which suggests that NSSI is proportionally more common among males in Asia than in Western contexts, provides a strong justification for our sex-stratified analysis. Importantly, the lack of conceptual clarity regarding female gender as a risk factor may not stem solely from inconsistencies in prevalence or phenotypic expression (e.g., methods of self-injury) [9,10] but rather from a relative dearth of research examining whether the underlying mechanisms contributing to the engagement and severity of NSSI differ between males and females. Our study thus aims to address this gap by elucidating the specific etiological pathways within this population.

To better understand these underlying mechanisms, extensive empirical work has sought to identify and integrate the key determinants of NSSI. An integrated theoretical model by Nock [11–13] suggests that NSSI is primarily reinforced by its functional consequences (e.g., affect regulation and self-punishment, and peer bonding), and the functions of NSSI develop when distal risk factors contribute to intrapersonal and interpersonal vulnerabilities. Furthermore, this model suggests that distal risk factors (e.g., childhood maltreatment), proximal stressors (e.g., current perceived stress), and emotional vulnerability (e.g., difficulties in emotion processing) are the principal contributors to both the engagement and severity of NSSI.

Early adverse interpersonal experiences, including childhood maltreatment [14] and peer-victimization [15], have been identified as a significant risk factor of NSSI. Among these, childhood maltreatment has been shown to exert a lasting influence on a wide range of adverse mental health outcomes, including NSSI [14,16–18]. However, the direct relationship between childhood maltreatment and NSSI appears less robust than previously assumed, with growing evidence suggesting that intermediary variables (e.g., depression and anxiety) may mediate this relationship [19–21]. Moreover, the effects of childhood maltreatment are often contingent on sample characteristics (e.g., males and females) and subtypes of maltreatment (i.e., emotional, physical, and sexual abuse, as well as emotional and physical neglect) [22–24]. Given that males and females, respectively, can be differentially associated with specific forms of maltreatment [25,26], it is necessary to consider differential pathways between males and females to NSSI engagement. Indeed, Serafini and Canepa [27] highlighted potential differences between males and females in the impact of childhood trauma on NSSI and suicidal behaviors, although further investigation is warranted to expand on these findings, which were based on relatively small samples.

In addition to distal factors, proximal distress plays a significant role in NSSI. A meta-analysis by Liu and Cheek [28] identified a small but statistically significant relationship between proximal life stress and NSSI. This association has been substantiated through ecological momentary assessment (EMA) studies, which demonstrate that daily fluctuations in stress levels significantly predict NSSI engagement on a given day [29,30]. Similarly, daily diary studies have found that higher levels of current perceived stress are associated with increased likelihood of NSSI thoughts [31]. Despite these findings, there remains a paucity of research examining how distal and proximal risk factors might jointly influence NSSI behaviors, particularly within sex-stratified models.

The bulk of the literature suggests that individuals who engage in NSSI may have difficulty processing emotions [32,33]. Alexithymia, characterized by the inability to identify and describe one’s feelings, has demonstrated its transdiagnostic role in maladaptive psychological experiences, such as childhood maltreatment [34], as well as NSSI [33]. Regarding NSSI, it suggests that the initiation stage of emotion processing may be disrupted in individuals with NSSI. Numerous studies have indicated that individuals with a history of NSSI exhibit significantly higher levels of alexithymia, suggesting that it may serve as a substantial predictor of NSSI [35–39] and contribute to deficits in emotion regulation [40]. However, compared to studies consistently reporting a strong connection between alexithymia and NSSI among females, research involving malesen is relatively limited and yields mixed results [41]. A recent meta-analysis identified a significantly larger effect size in the relationship between alexithymia and self-harm in females than in males, noting the dearth of studies that have separately investigated males and females within the research samples [42]. Moreover, given the predominantly female participant data in previous studies, it is crucial to examine the potential sex differences in the association between alexithymia and NSSI.

Accordingly, we hypothesized that childhood maltreatment, current perceived stress, and alexithymia would each be significantly associated with the likelihood of engaging in NSSI, with these associations exhibiting sex-specific patterns. We further examined the extent to which these clinical variables were related to NSSI severity, operationalised through method versatility, by conducting sex-stratified regression analyses. Specifically, we hypothesized that subtypes of childhood maltreatment would be associated with an increased risk of NSSI engagement and severity, but that distinct subtypes of childhood maltreatment would be uniquely associated with males and females, respectively. We also hypothesized that current perceived stress would be significantly associated with both NSSI engagement and severity in both males and females. Finally, we hypothesized that alexithymia would be significantly associated with NSSI engagement and severity in females, but not in males. Through this multidimensional, sex-informed framework, the study seeks to elucidate the mechanisms underlying NSSI and thereby contribute to developing more tailored and effective prevention and intervention strategies.

## Materials and methods

### Participants and procedures

We recruited 835 adults (ages 19–29) fluent in written Korean through online forums and social media platforms. The survey was available for five months, from October 18, 2018 to March 9, 2019. Recruitment advertisements explicitly informed potential participants that the study concerned risk factors related to NSSI and aimed to enroll individuals with and without a history of NSSI to ensure an informed consent process. Following the acquisition of informed consent, participants completed a yes-no forced-choice question regarding whether they had engaged in five or more instances of NSSI in their lifetime. They subsequently completed psychometric questionnaires, reported any history of suicide attempts, and reported demographic data, including sex assigned at birth, age, and educational attainment. All survey questions were administered in Korean. The inclusion criterion was fluency in Korean. Participants were recruited regardless of their lifetime history of NSSI and subsequently classified into two groups: (1) those with a history of NSSI during their lifetime, and (2) those without a history of NSSI during their lifetime.

From the initial sample, 104 participants were excluded for the following reasons: self-harm behaviors did not meet NSSI criteria (*n* = 43), patterned or otherwise unreliable responses (*n* = 58), or a history of suicide attempts without NSSI (*n* = 3). The final analytic sample comprised 731 participants. Participants were divided into two groups: the NSSI group (124 males and 357 females), defined as individuals who reported five or more lifetime NSSI behaviors, and the Control group (109 males and 141 females), indicating those who reported neither a history of lifetime NSSI behaviors nor suicide attempts. Participants who reported a history of suicide attempts without NSSI were excluded due to the small sample size. The study received ethical approval from the Institutional Review Board (1041078–201706-BRSB-127-0C). All participants provided written informed consent prior to data collection.

### Measures

#### NSSI.

The versatility of the NSSI method was assessed using Section I of the Korean version of the Inventory of Statements about Self-injury (ISAS) [43,44]. Section I comprises a behavioral checklist of self-injurious behaviors, for which participants report the frequency of each behavior. In the present study, method versatility was operationalized as a cumulative count, determined by summing the presence of each distinct self-injurious behavior (scored as 1 if present, 0 if absent). This indicator has been validated in prior research, which has shown that greater method versatility is associated with more severe psychopathology and increased suicide risk [45–47]. Additionally, the age of NSSI onset was obtained from Section I of the ISAS, which requires participants to report the date of their first engagement in NSSI.

#### Childhood maltreatment.

Childhood maltreatment was measured using the Childhood Trauma Questionnaire (CTQ) [48,49], a 28-item self-administered questionnaire evaluating five subscales: emotional abuse, physical abuse, sexual abuse, emotional neglect, and physical neglect. Responses are rated on a five-point Likert scale (1 = *never true*; 2 = *rarely true*; 3 = *sometimes true*; 4 = *often true*; and 5 = *very often true*). In the current study, the internal consistency of the CTQ was good (Cronbach’s *α* = .87).

#### Current perceived stress.

Current stress over the past month was assessed using the 10-item Perceived Stress Scale (PSS) [50,51], rated on a five-point Likert scale (0 = *never*, 1 = *rarely*, 2 = *sometimes*, 3 = *often*, 4 = *very often*). The PSS demonstrated good internal consistency (*α* = .89) in this study.

#### Alexithymia.

Alexithymia was measured using the 20-item Toronto Alexithymia Scale (TAS) [52], including subscales for difficulty identifying feelings (DIF), difficulty describing feelings (DDF), and externally oriented thinking (EOT). Responses are reported on a five-point Likert scale (1 = *strongly disagree* to 5 = *strongly agree*). The Korean version of the TAS-20 [53] demonstrated good internal consistency (*α* = .88) in this study. For subscales, DIF (*α* = .90) and DDF (*α* = .80) both showed good internal consistency. However, consistent with previous research [54–56], the internal consistency for EOT was unacceptable in the present study (*α* = .50).

### Statistical analyses

All data analyses were conducted using SPSS version 29 (IBM Corporation, Armonk, NY, USA). Group differences were assessed using one-way analysis of variance (ANOVA) for continuous variables and *chi-*square tests for categorical variables. ANOVA results were reported along with Bonferroni post-hoc tests to control for multiple comparisons. The effect size of group differences was reported using partial *η*^2^ for ANOVA.

Binary logistic regression was performed to identify clinical variables (CTQ subtypes, PSS, and TAS scores) as predictors of NSSI engagement in the total sample, controlling for sex (female = 1, male = 0, used as the reference group). Next, for a sex-stratified analysis, binary logistic regression was used within each sex group. NSSI engagement was defined as whether they engaged five or more times in lifetime NSSI (if yes = 1, no = 0). For participants in the NSSI group, multiple regression analyses were performed separately for males and females, with NSSI versatility as the dependent variable and CTQ, PSS, and TAS scores as independent variables. The statistical significance level was set at *P* < .05.

## Results

### Sociodemographic characteristics

Among the 731 participants, 498 (68.13%) were females, with a mean age of 22.61 years (*SD* = 2.67). For sex-specific analysis, we divided participants into four groups: males who engaged in NSSI (*n* = 124), females who engaged in NSSI (*n* = 357), males without a history of NSSI or suicide attempts (*n* = 109), and females without a history of NSSI or suicide attempts (*n* = 141). Significant age differences were observed across the four groups (*F*_(3, 727)_ = 3.40, *P* = .017), with Bonferroni’s post hoc test revealing that males in the NSSI group were older than females in the NSSI group. No significant differences in educational attainment were found among the groups (*χ*^2^_(9)_ = 12.84, *P* = .170), as shown in Table 1. The age of onset of NSSI did not differ between males and females in the NSSI group (*F*_(1, 477)_ = 0.05, partial *η*^2^ = .00, *P* = .828). However, females reported significantly greater NSSI versatility than males (*F*_(1, 479)_ = 21.89, partial *η*^2^ = .04, *P* < .001).

**Table 1:** Sociodemographic and clinical characteristics (*N* = 731).

	Males		Females		*F/(χ*^2^)	*P*
	NSSI (*n* = 124)	Control (*n* = 109)	NSSI (*n* = 357)	Control (*n* = 141)		
Age	23.17 ± 2.71	22.88 ± 2. 65	22.34 ± 2.73	22.57 ± 2.45	3.40	.017^a^
Education attainment					(12.84)	.170
< High school degree	1 (0.8%)	0 (0%)	1 (0.3%)	0 (0%)		
High school degree	12 (9.7%)	10 (9.2%)	54 (15.1%)	10 (7.1%)		
Currently in college	80 (64.5%)	79 (72.5%)	217 (60.8%)	94 (66.7%)		
≥ College degree	31 (25.0%)	20 (18.3%)	85 (23.8%)	37 (26.2%)		
Age of onset for NSSI	14.58 ± 6.02		14.46 ± 4.98		0.05	.828
NSSI versatility	4.51 ± 2.52		5.80 ± 2.70		21.89	<.001
CTQ						
Emotional abuse	8.95 ± 4.01	7.27 ± 3.14	12.73 ± 5.33	8.08 ± 3.30	66.38	<.001^b, c, d, e^
Physical abuse	10.47 ± 5.34	7.54 ± 3.44	10.97 ± 5.66	7.87 ± 4.07	21.33	<.001^b, d, e, f^
Sexual abuse	6.21 ± 2.63	5.93 ± 2.36	6.95 ± 3.78	5.72 ± 1.84	6.94	<.001^d, e^
Emotional neglect	11.31 ± 5.23	8.69 ± 4.30	14.85 ± 5.88	10.06 ± 4.60	53.68	<.001^b, c, d, e^
Physical neglect	8.90 ± 3.57	7.39 ± 3.20	9.55 ± 4.06	7.23 ± 2.51	19.54	<.001^b, d, e, f^
PSS	19.84 ± 6.57	15.85 ± 7.38	24.70 ± 7.32	17.99 ± 7.19	59.35	<.001^b, c, d, e^
TAS	50.10 ± 11.39	44.60 ± 11.08	57.40 ± 13.01	45.50 ± 11.34	51.38	<.001^b, c, d, e, f^

NSSI, Nonsuicidal self-injury; CTQ, Childhood Trauma Questionnaire; PSS, Perceived Stress Scale; TAS, Toronto Alexithymia Scale.

^a^ NSSI males > NSSI females; ^b^ NSSI males > control males; ^c^ NSSI females > NSSI males; ^d^ NSSI females > control males; ^e^ NSSI females > control females; ^f^ NSSI males > control females.

### Comparison of NSSI and control groups: clinical characteristics

Significant group differences were found across all five subtypes of childhood trauma: emotional abuse (*F*_(3, 727)_ = 66.38, partial *η*^2^ = .22, *P* *<* .001), physical abuse (*F*_(3, 727)_ = 21.33, partial *η*^2^ = .08, *P* *<* .001), sexual abuse (*F*_(3, 727)_ = 6.94, partial *η*^2^ = .03, *P* *<* .001), emotional neglect (*F*_(3, 727)_ = 53.68, partial *η*^2^ = .18, *P* *<* .001), and physical neglect (*F*_(3, 727)_ = 19.54, partial *η*^2^ = .08, *P* *<* .001; Table 1). Bonferroni’s post hoc analyses revealed that for emotional abuse and emotional neglect, both males and females in the NSSI group demonstrated significantly higher levels of emotional abuse and emotional neglect than the control group, and females in the NSSI group reported significantly higher levels than males in the NSSI group. Regarding physical abuse and physical neglect, both males and females in the NSSI group had significantly higher levels than the control group. However, no significant differences were found between males and females in the NSSI group. For sexual abuse, females in the NSSI group reported significantly higher levels of experiences compared to the control group. However, no significant differences were found between NSSI males and NSSI females, nor between NSSI males and the control group.

Significant differences in current perceived stress were also identified among the four groups (*F*_(3, 727)_ = 59.35, partial *η*^2^ = .20, *P* < .001). Post hoc comparisons indicated that both males and females in the NSSI group demonstrated significantly higher stress levels than those in the control group, and females in the NSSI group reported significantly higher stress levels than males in the NSSI group.

Regarding alexithymia, significant group differences were found (*F*_(3, 727)_ = 51.38, partial *η*^2^ = .18, *P* < .001). Post hoc analyses showed that both males and females in the NSSI group demonstrated significantly higher alexithymia levels than those in the control group. Females in the NSSI group reported significantly higher alexithymia levels than males in the NSSI group.

### Effects of clinical variables on NSSI engagement for each sex group

In the total sample, bivariate logistic regression analyses (see Table 2) revealed that emotional abuse (odds ratio [OR] = 1.10, *P* = .008), current perceived stress (OR = 1.05, *P* = .003), and alexithymia (OR = 1.03, *P* = .001) were all significantly associated with increased odds of NSSI engagement. Sex did not demonstrate a significant association with odds of NSSI engagement (OR = 1.20, *P* = .343). The overall logistic regression model was statistically significant (*χ*^2^_(7)_ = 186.5, *P* < .001), with Nagelkerke *R*² indicating that the model explained 30.8% of the variance (Nagelkerke *R*² = .308).

**Table 2:** Bivariate logistic regression analyses on NSSI engagement for all participants (*N* = 731).

	Total (*N* = 731)		
	OR	CI	*P*
Sex (Female)	1.20	0.82-1.76	.343
CTQ			
Emotional abuse	**1.10**	**1.02–1.19**	**.008**
Physical abuse	1.03	0.98–1.09	.237
Sexual abuse	1.00	0.93–1.08	.979
Emotional neglect	1.02	0.97–1.08	.415
Physical neglect	1.02	0.95–1.11	.543
PSS	**1.05**	**1.02–1.08**	**.003**
TAS	**1.03**	**1.01–1.05**	**.001**

NSSI, Nonsuicidal Self-injury; OR, Odds Ratio; CI, Confidence Interval; CTQ, Childhood Trauma Questionnaire; PSS, Perceived Stress Scale; TAS, Toronto Alexithymia Scale.

Separate logistic regression models were conducted for males and females, respectively (Table 3). Among males, only physical abuse emerged as a significant predictor of NSSI engagement. Specifically, males with a history of physical abuse were 1.19 times more likely to engage in NSSI compared to those without such experiences (OR = 1.19, *P* = .001). The association between current stress and NSSI presence was not significant (OR = 1.05, *P* = .079), and alexithymia was not a significant predictor in males (OR = 1.02, *P* = .373). The regression model was significant (*χ*^2^_(7)_ = 39.3, *P* < .001), and explained 20.7% of the variance (Nagelkerke *R*² = .207).

**Table 3:** Sex-stratified bivariate logistic regression analyses on NSSI engagement for all participants (*N* = 731).

	Males (*n* = 233)			Females (*n* = 498)		
	OR	CI	*P*	OR	CI	*P*
CTQ						
Emotional abuse	0.89	0.77–1.03	.120	**1.19**	**1.08–1.30**	**<.001**
Physical abuse	**1.19**	**1.07–1.32**	**.001**	0.98	0.91–1.05	.488
Sexual abuse	0.93	0.81–1.06	.246	1.05	0.95–1.16	.345
Emotional neglect	1.06	0.96–1.17	.233	1.00	0.93–1.07	.983
Physical neglect	1.04	0.92–1.17	.547	1.05	0.95–1.16	.341
PSS	1.05	1.00–1.10	.079	**1.05**	**1.01–1.09**	**.016**
TAS	1.02	0.98–1.05	.373	**1.04**	**1.02–1.07**	**<.001**

NSSI, Nonsuicidal Self-injury; OR, Odds Ratio; CI, Confidence Interval; CTQ, Childhood Trauma Questionnaire; PSS, Perceived Stress Scale; TAS, Toronto Alexithymia Scale.

Among females, emotional abuse was associated with a 1.19-fold risk for engaging in NSSI compared to those without such experiences (OR = 1.19, *P* < .001). Additionally, each unit increase in current stress was associated with a 5% increase in the odds of NSSI (OR = 1.05, *P* = .016), and each unit increase in alexithymia was associated with a 4% increase in odds (OR = 1.04, *P* < .001). This regression model was statistically significant (*χ*^2^_(7)_ = 143.9, *P* < .001), with Nagelkerke *R*² indicating 36% of the variance explained (Nagelkerke *R*² = .360).

### Severity in the NSSI group

Multiple regression analyses were conducted to investigate predictors of NSSI method versatility among individuals in the NSSI group (Table 4). For the total NSSI sample, emotional abuse (*β* = .15, *t* = 2.10, *P* = .036) and sexual abuse (*β* = .13, *t* = 3.00, *P* = .003) significantly predicted greater method versatility. In addition, current perceived stress (*β* = .14, *t* = 2.53, *P* = .012) and alexithymia (*β* = .20, *t* = 3.78, *P* < .001) were also significant predictors. The overall model was significant (*F*_(7, 473)_ = 17.15, *P* < .001, Adjusted *R*^2^ = 0.19), explaining 19% of the variance.

**Table 4:** Multiple regression analyses on the versatility of NSSI by sex for the NSSI group (*n* = 481).

	NSSI Total (*n* = 481)			Males engaging in NSSI (*n* = 124)			Females engaging in NSSI (*n* = 357)		
	β	*t*	*P*	β	*t*	*P*	β	*t*	*P*
CTQ									
Emotional abuse	**.15**	**2.10**	**.036**	.02	0.11	.914	.14	1.78	.076
Physical abuse	.01	0.15	.879	.17	1.51	.134	−.02	−0.32	.752
Sexual abuse	**.13**	**3.00**	**.003**	.15	1.73	.087	**.13**	**2.54**	**.011**
Emotional neglect	−.06	−0.91	.362	.05	0.31	.757	−.10	−1.30	.197
Physical neglect	.09	1.61	.108	−.15	−1.25	.213	**.18**	**2.69**	**.008**
PSS	**.14**	**2.53**	**.012**	**.24**	**2.46**	**.015**	.08	1.34	.181
TAS	**.20**	**3.78**	**<.001**	.17	1.65	.101	**.20**	**3.36**	**.001**

NSSI, Nonsuicidal Self-injury; CTQ, Childhood Trauma Questionnaire; PSS, Perceived Stress Scale; TAS, Toronto Alexithymia Scale.

#### Males engaging in NSSI.

None of the childhood trauma subtypes significantly predicted NSSI versatility in males (all *P* > .05). Current perceived stress was a significant predictor of NSSI versatility in males (*β* = .24, *t* = 2.46, *P* = .015), while alexithymia was not (*P* = .101). The regression model was significant (*F*_(7, 116)_ = 4.20, *P* < .001), explaining 15% of the variance (Adjusted *R*^2^ = 0.15).

#### Females engaging in NSSI.

Sexual abuse (*β* = .13, *t* = 2.54, *P* = .011) and physical neglect (*β* = .18, *t* = 2.69, *P* = .008) significantly predicted the NSSI versatility in females. Current perceived stress was not a significant predictor (*P* = .181). In contrast, alexithymia emerged as a significant predictor (*β* = .20, *t* = 3.36, *P* = .001). The regression model was statistically significant (*F*_(7, 349)_ = 11.28, *P* < .001, Adjusted *R*^2^ = 0.17), explaining 17% of the variance.

## Discussion

The current study found that subtypes of childhood maltreatment, current stress, and alexithymia demonstrated distinct, sex-specific associations with both the engagement and severity of NSSI. Specifically, in males, physical abuse emerged as a significant predictor of NSSI engagement, while current stress was uniquely related to greater NSSI method versatility. Conversely, in females, emotion abuse, current stress, and alexithymia were each independently associated with an increased risk of NSSI, while sexual abuse, physical neglect, and alexithymia predicted greater severity. These results critically underscore the existence of sex-specific pathways underpinning NSSI engagement and severity, which indicates that tailoring assessment and intervention strategies to account for these divergent patterns may be required.

Consistent with our hypothesis, subtypes of childhood maltreatment were associated with increased risk of NSSI engagement and severity, but distinct subtypes of maltreatment were uniquely associated with males and females, respectively. Our findings of higher overall childhood maltreatment in the NSSI group compared to controls reinforce the established role of early adversity as a distal risk factor [57,58]. Importantly, our sex-stratified analysis advances the field by revealing sex-specific associations between maltreatment types and NSSI, addressing inconsistencies in prior research [20,23,59]. Although problems regarding statistical power due to differences in sample size should be noted, in line with previous studies [60–62], physical abuse was exclusively related to the NSSI engagement in males. No discernible relationship was found between childhood sexual abuse and the NSSI engagement in either sex, consistent with prior studies reporting weak [63], indirect [20], or absent correlations [23,62,64,65]. However, among females, both sexual abuse and physical neglect were associated with greater NSSI severity. These results align with previous findings in predominantly female samples [43,66], suggesting that while sexual abuse may not directly initiate NSSI, it may exacerbate its severity. Prior work has proposed that emotion dysregulation [67], social avoidance [68], and lack of social supports [69] mediate this association.

Additionally, females engaging in NSSI reported higher levels of emotional abuse and neglect compared to both males engaging in NSSI and controls. Further, among females, emotional abuse was the most salient predictor of NSSI, echoing results from large clinical samples [70] and adolescent research with predominantly female participants (77.7%) [71]. Emotional abuse has been linked to emotion dysregulation [72] and maladaptive cognitive styles such as rumination [73], both of which are known contributors to NSSI in females. These findings suggest that difficulties in emotional processing may serve as mechanisms linking emotional abuse to self-injury in females, given that emotional abuse may influence the maintenance of NSSI in adolescence [74]. Our results underscore the critical importance of disentangling the influence of each subtype of childhood maltreatment. This specificity is crucial, as it suggests that a detailed assessment of maltreatment history is fundamentally required to develop effective and tailored clinical approaches for individuals engaging in NSSI.

With respect to proximal stress, females with NSSI reported higher perceived stress levels than their control counterparts. However, stress was not a significant predictor of NSSI severity, contrary to our hypothesis. In males, by contrast, current stress was associated with NSSI severity, despite not being elevated relative to the control group. These findings are consistent with studies reporting weak associations between stress and NSSI severity [75–77], suggesting that the effect of stress on NSSI may be more closely tied to the exacerbation of existing behaviors rather than the incidence of NSSI. Consistently, a longitudinal study [78] has reported that stressful events significantly increase the risk of NSSI through emotion dysregulation over time, indicating that NSSI is not merely an immediate reaction to stressful events, but rather an outcome of the interplay between stressful experiences and deficits in emotion regulation. Further studies need to elucidate the mechanisms by which current stress is associated with increased NSSI engagement and severity within each sex, particularly by examining the mediating role of emotion dysregulation. Our findings emphasize the importance of considering individual and sex-related differences when assessing the impact of proximal stressors.

As hypothesized, alexithymia also emerged as a robust predictor of both the engagement and severity of NSSI in females, which aligns with previous studies [36–38,79]. Substantial work supports the pathway where childhood maltreatment contributes to the development of alexithymic traits, which in turn mediates the relationship between early adversity and later psychopathology [80]. This consistent finding suggests that, for females, the poor emotional cognition inherent in alexithymia directly increases vulnerability to NSSI, supporting an affect regulation model where self-harm is used to manage poorly understood emotional states [81,82]. For instance, one adolescent study found a significant association between alexithymia and NSSI frequency in girls but not boys [83], a finding confirmed by a recent meta-analysis [39] showing stronger associations in female-only samples. In contrast, the absence of a significant predictive link in our male sample aligns with a subset of previous studies that found inconclusive or non-significant associations between alexithymia and self-harm in males [6,42]. This pattern suggests that, for males, emotional distress leading to NSSI may be mediated by factors other than core alexithymic traits, potentially involving more external motivations or behaviors as a form of expression [82,84]. Therefore, clinicians assessing clients should not rely solely on alexithymia scores, but rather consider sex differences in emotion recognition and processing to determine tailored interventions. These may include emotion regulation skills training for females and exploring the function of externalized behaviors for males [70,85,86].

Despite these crucial findings, several limitations of this research should be noted. First, the current study is constrained by its cross-sectional, retrospective design, which prevents the establishment of causality; thus, longitudinal studies are required to confirm the temporal direction of effects, particularly concerning current stress experiences. Relatedly, the reliance on retrospective self-reported data for childhood maltreatment introduces potential recall bias, and while subjective experiences of adversity may better predict psychopathology [87,88], future research should examine the discrepancies with objective reports. Furthermore, as a cross-sectional investigation, we did not apply explicit criteria for ‘clinical stability’ (e.g., stable medication or absence of an acute crisis) during recruitment, meaning the influence of rapid symptom fluctuation on the reported associations cannot be fully excluded. Also, the lack of information regarding the recency of NSSI behaviors should be addressed as a limitation. Secondly, sample limitations affect the generalizability of the findings. The measure of sex reflects the biological sex assigned at birth and fails to capture the social dimensions of gender [89,90], thereby restricting generalization to individuals whose gender identity differs from their biological sex. Given that sexual and gender minorities are associated with an increased risk of NSSI [91,92], their exclusion further restricts applicability. Additionally, the relatively small sample size of males limits the generalizability of the findings, as the results for the entire NSSI group (*n* = 481) largely mirrored those for females (*n* = 357), likely due to the statistical power in the male subsample. Finally, psychometric limitations must be noted: our measure of alexithymia (TAS-20) exhibited limited internal consistency in one subscale (EOT, *α* = .50). Crucially, the absence of a measure for depressive symptomatology, a known confounder of TAS scores [93–95], means its influence could not be statistically controlled, potentially leading to an overestimation of the unique effect of alexithymia. This measurement limitation also applies to the interpretation of severity: the observed higher NSSI method versatility in females suggests a disparity in the perceived effectiveness of the behavior for achieving NSSI functions [96]. Further research should explicitly investigate these functions and their perceived effectiveness to elucidate these sex-specific mechanisms.

This study established that childhood maltreatment, current stress, and alexithymia contribute to NSSI through qualitatively distinct sex-specific etiological pathways. This underscores the necessity of moving beyond sole risk factor assessment toward a detailed, tailored evaluation of these factors, especially the underlying emotional processing deficits that drive NSSI behaviors, to inform intervention strategies. Specifically, effective interventions should target emotional awareness and expression for females, which may mitigate the effects of emotional abuse and alexithymia, while stress management strategies are recommended for males to address NSSI severity. Further research should prioritize exploring sex-specific trajectories of emotion regulation to elucidate these divergent pathways fully.

## References

[1] Swannell SV, Martin GE, Page A, Hasking P, St John NJ. Prevalence of nonsuicidal self-injury in nonclinical samples: systematic review, meta-analysis and meta-regression. Suicide Life Threat Behav. 2014;44(3):273–303. doi: 10.1111/sltb.12070 24422986

[2] Kiekens G, Hasking P, Bruffaerts R, Alonso J, Auerbach RP, Bantjes J, et al. Non-suicidal self-injury among first-year college students and its association with mental disorders: results from the World Mental Health International College Student (WMH-ICS) initiative. Psychol Med. 2023;53(3):875–86. doi: 10.1017/S0033291721002245 34140062 PMC8683565

[3] Ribeiro JD, Franklin JC, Fox KR, Bentley KH, Kleiman EM, Chang BP, et al. Self-injurious thoughts and behaviors as risk factors for future suicide ideation, attempts, and death: a meta-analysis of longitudinal studies. Psychol Med. 2016;46(2):225–36. doi: 10.1017/S0033291715001804 26370729 PMC4774896

[4] Calvo N, García-González S, Perez-Galbarro C, Regales-Peco C, Lugo-Marin J, Ramos-Quiroga J-A, et al. Psychotherapeutic interventions specifically developed for NSSI in adolescence: A systematic review. Eur Neuropsychopharmacol. 2022;58:86–98. doi: 10.1016/j.euroneuro.2022.02.009 35325633

[5] Pompili M, Baldessarini RJ, Forte A, Erbuto D, Serafini G, Fiorillo A, et al. Do atypical antipsychotics have antisuicidal effects? A hypothesis-generating overview. Int J Mol Sci. 2016;17(10):1700. doi: 10.3390/ijms17101700 27727180 PMC5085732

[6] Bresin K, Schoenleber M. Gender differences in the prevalence of nonsuicidal self-injury: A meta-analysis. Clin Psychol Rev. 2015;38:55–64. doi: 10.1016/j.cpr.2015.02.009 25795294

[7] Moloney F, Amini J, Sinyor M, Schaffer A, Lanctôt KL, Mitchell RHB. Sex differences in the global prevalence of nonsuicidal self-injury in adolescents: a meta-analysis. JAMA Netw Open. 2024;7(6):e2415436. doi: 10.1001/jamanetworkopen.2024.15436 38874927 PMC11179134

[8] Farkas BF, Takacs ZK, Kollárovics N, Balázs J. The prevalence of self-injury in adolescence: a systematic review and meta-analysis. Eur Child Adolesc Psychiatry. 2024;33(10):3439–58. doi: 10.1007/s00787-023-02264-y 37486387 PMC11564408

[9] Andover MS, Primack JM, Gibb BE, Pepper CM. An examination of non-suicidal self-injury in men: Do men differ from women in basic NSSI characteristics?. Arch Suicide Res. 2010;14(1):79–88. doi: 10.1080/13811110903479086 20112146

[10] Sornberger MJ, Heath NL, Toste JR, McLouth R. Nonsuicidal self-injury and gender: patterns of prevalence, methods, and locations among adolescents. Suicide Life Threat Behav. 2012;42(3):266–78. doi: 10.1111/j.1943-278X.2012.0088.x 22435988

[11] Nock MK. Self-injury. Annu Rev Clin Psychol. 2010;6:339–63. doi: 10.1146/annurev.clinpsy.121208.131258 20192787

[12] Nock MK. Why do people hurt themselves? New insights into the nature and functions of self-injury. Curr Dir Psychol Sci. 2009;18(2):78–83. doi: 10.1111/j.1467-8721.2009.01613.x 20161092 PMC2744421

[13] Fox KR, Franklin JC, Ribeiro JD, Kleiman EM, Bentley KH, Nock MK. Meta-analysis of risk factors for nonsuicidal self-injury. Clin Psychol Rev. 2015;42:156–67. doi: 10.1016/j.cpr.2015.09.002 26416295 PMC4772426

[14] Calvo N, Lugo-Marín J, Oriol M, Pérez-Galbarro C, Restoy D, Ramos-Quiroga J-A, et al. Childhood maltreatment and non-suicidal self-injury in adolescent population: A systematic review and meta-analysis. Child Abuse Negl. 2024;157:107048. doi: 10.1016/j.chiabu.2024.107048 39332140

[15] Serafini G, Aguglia A, Amerio A, Canepa G, Adavastro G, Conigliaro C, et al. The relationship between bullying victimization and perpetration and non-suicidal self-injury: a systematic review. Child Psychiatry Hum Dev. 2023;54(1):154–75. doi: 10.1007/s10578-021-01231-5 34435243 PMC9867675

[16] Teicher MH, Gordon JB, Nemeroff CB. Recognizing the importance of childhood maltreatment as a critical factor in psychiatric diagnoses, treatment, research, prevention, and education. Mol Psychiatry. 2022;27(3):1331–8. doi: 10.1038/s41380-021-01367-9 34737457 PMC8567985

[17] Erol Y, Inozu M. An investigation of the mediating roles of emotion regulation difficulties, distress tolerance, self-compassion, and self-disgust in the association between childhood trauma and nonsuicidal self-injury. Arch Suicide Res. 2024;28(3):815–29. doi: 10.1080/13811118.2023.2237083 37470456

[18] Xie X, Li Y, Liu J, Zhang L, Sun T, Zhang C, et al. The relationship between childhood maltreatment and non-suicidal self-injury in adolescents with depressive disorders. Psychiatry Res. 2024;331:115638. doi: 10.1016/j.psychres.2023.115638 38035534

[19] Gong X, Zhang L. Childhood Maltreatment and Non-Suicidal Self-Injury in Adolescents: Testing a Moderated Mediating Model. J Interpers Violence. 2024;39(5–6):925–48. doi: 10.1177/08862605231197747 38229266

[20] Brown RC, Heines S, Witt A, Braehler E, Fegert JM, Harsch D, et al. The impact of child maltreatment on non-suicidal self-injury: data from a representative sample of the general population. BMC Psychiatry. 2018;18(1):181. doi: 10.1186/s12888-018-1754-3 29884152 PMC5994090

[21] Zhang M, Liu X, Xia W, Zhang W, Yu H, Ma H, et al. Network analysis of childhood maltreatment, anxiety, and addictive non-suicidal self-injury in adolescents. Int J Ment Health Addiction. 2024;23(6):4178–90. doi: 10.1007/s11469-024-01344-7

[22] Muehlenkamp JJ, Claes L, Smits D, Peat CM, Vandereycken W. Non-suicidal self-injury in eating disordered patients: a test of a conceptual model. Psychiatry Res. 2011;188(1):102–8. doi: 10.1016/j.psychres.2010.12.023 21216476

[23] Thomassin K, Shaffer A, Madden A, Londino DL. Specificity of childhood maltreatment and emotion deficit in nonsuicidal self-injury in an inpatient sample of youth. Psychiatry Res. 2016;244:103–8. doi: 10.1016/j.psychres.2016.07.050 27479099

[24] Rabinovitch SM, Kerr DCR, Leve LD, Chamberlain P. Suicidal behavior outcomes of childhood sexual abuse: longitudinal study of adjudicated girls. Suicide Life Threat Behav. 2015;45(4):431–47. doi: 10.1111/sltb.12141 25370436 PMC4420727

[25] Christoffersen MN, Armour C, Lasgaard M, Andersen TE, Elklit A. The prevalence of four types of childhood maltreatment in denmark. Clin Pract Epidemiol Ment Health. 2013;9:149–56. doi: 10.2174/1745017901309010149 24155769 PMC3804885

[26] Wan Y, Chen J, Sun Y, Tao F. Impact of childhood abuse on the risk of non-suicidal self-injury in mainland chinese adolescents. PLoS One. 2015;10(6):e0131239. doi: 10.1371/journal.pone.0131239 26114574 PMC4482708

[27] Serafini G, Canepa G, Adavastro G, Nebbia J, Belvederi Murri M, Erbuto D, et al. The relationship between childhood maltreatment and non-suicidal self-injury: a systematic review. Front Psychiatry. 2017;8:149. doi: 10.3389/fpsyt.2017.00149 28970807 PMC5609590

[28] Liu RT, Cheek SM, Nestor BA. Non-suicidal self-injury and life stress: A systematic meta-analysis and theoretical elaboration. Clin Psychol Rev. 2016;47:1–14. doi: 10.1016/j.cpr.2016.05.005 27267345 PMC4938721

[29] Miller AB, Eisenlohr-Moul T, Glenn CR, Turner BJ, Chapman AL, Nock MK, et al. Does higher-than-usual stress predict nonsuicidal self-injury? Evidence from two prospective studies in adolescent and emerging adult females. J Child Psychol Psychiatry. 2019;60(10):1076–84. doi: 10.1111/jcpp.13072 31054205 PMC6953610

[30] Kleiman EM, Turner BJ, Chapman AL, Nock MK. Fatigue moderates the relationship between perceived stress and suicidal ideation: evidence from two high-resolution studies. J Clin Child Adolesc Psychol. 2018;47(1):116–30. doi: 10.1080/15374416.2017.1342543 28715280

[31] Turner BJ, Baglole JS, Chapman AL, Gratz KL. Experiencing and resisting nonsuicidal self-injury thoughts and urges in everyday life. Suicide Life Threat Behav. 2019;49(5):1332–46. doi: 10.1111/sltb.12510 30152181

[32] Wolff JC, Thompson E, Thomas SA, Nesi J, Bettis AH, Ransford B, et al. Emotion dysregulation and non-suicidal self-injury: A systematic review and meta-analysis. Eur Psychiatry. 2019;59:25–36. doi: 10.1016/j.eurpsy.2019.03.004 30986729 PMC6538442

[33] Iskric A, Ceniti AK, Bergmans Y, McInerney S, Rizvi SJ. Alexithymia and self-harm: A review of nonsuicidal self-injury, suicidal ideation, and suicide attempts. Psychiatry Res. 2020;288:112920. doi: 10.1016/j.psychres.2020.112920 32279008

[34] Ditzer J, Wong EY, Modi RN, Behnke M, Gross JJ, Talmon A. Child maltreatment and alexithymia: A meta-analytic review. Psychol Bull. 2023;149(5–6):311–29. doi: 10.1037/bul0000391 37261746

[35] Tang WC, Lin MP, You J. et al. Prevalence and psychosocial risk factors of nonsuicidal self-injury among adolescents during the COVID-19 outbreak. Curr Psychol. 2023;42:17270–9. doi: 10.1007/s12144-021-01931-0 PMC8167308 34092987

[36] Lüdtke J, In-Albon T, Michel C, Schmid M. Predictors for DSM-5 nonsuicidal self-injury in female adolescent inpatients: The role of childhood maltreatment, alexithymia, and dissociation. Psychiatry Res. 2016;239:346–52. doi: 10.1016/j.psychres.2016.02.026 27088878

[37] Lambert A, de Man AF. Alexithymia, depression, and self-mutilation in adolescent girls. N Am J Psychol. 2007;9(3):555–66.

[38] Bordalo F, Carvalho IP. The role of alexithymia as a risk factor for self-harm among adolescents in depression - A systematic review. J Affect Disord. 2022;297:130–44. doi: 10.1016/j.jad.2021.10.029 34695502

[39] Greene D, Boyes M, Hasking P. The associations between alexithymia and both non-suicidal self-injury and risky drinking: A systematic review and meta-analysis. J Affect Disord. 2020;260:140–66. doi: 10.1016/j.jad.2019.08.088 31494366

[40] Baumann N, Ecker A, Schleicher D, Kandsperger S, Preece DA, Brunner R, et al. The relationship between alexithymia, non-suicidal self-injury, and emotion regulation. J Affect Disord. 2025;384:60–8. doi: 10.1016/j.jad.2025.05.009 40334863

[41] Norman H, Borrill J. The relationship between self-harm and alexithymia. Scand J Psychol. 2015;56(4):405–19. doi: 10.1111/sjop.12217 26011069

[42] Norman H, Oskis A, Marzano L, Coulson M. The relationship between self-harm and alexithymia: A systematic review and meta-analysis. Scand J Psychol. 2020;61(6):855–76. doi: 10.1111/sjop.12668 32706131

[43] Izdebska A. Personality Disorders in Adult Female Child Sexual Abuse Survivors: Dimensions of Personality Pathology and Characteristics of Abuse. J Interpers Violence. 2021;36(23–24):NP13487–516. doi: 10.1177/0886260520903136 32125218

[44] Klonsky ED, Glenn CR. Assessing the functions of non-suicidal self-injury: Psychometric properties of the Inventory of Statements About Self-injury (ISAS). J Psychopathol Behav Assess. 2009;31(3):215–9. doi: 10.1007/s10862-008-9107-z 29269992 PMC5736316

[45] Turner BJ, Layden BK, Butler SM, Chapman AL. How often, or how many ways: clarifying the relationship between non-suicidal self-injury and suicidality. Arch Suicide Res. 2013;17(4):397–415. doi: 10.1080/13811118.2013.802660 24224673

[46] Ammerman BA, Jacobucci R, Turner BJ, Dixon-Gordon KL, McCloskey MS. Quantifying the importance of lifetime frequency versus number of methods in conceptualizing nonsuicidal self-injury severity. Psychol Violence. 2020;10(4):442–51. doi: 10.1037/vio0000263

[47] Saraff PD, Pepper CM. Functions, lifetime frequency, and variety of methods of non-suicidal self-injury among college students. Psychiatry Res. 2014;219(2):298–304. doi: 10.1016/j.psychres.2014.05.044 24947915

[48] Bernstein DP, Stein JA, Newcomb MD, Walker E, Pogge D, Ahluvalia T, et al. Development and validation of a brief screening version of the Childhood Trauma Questionnaire. Child Abuse Negl. 2003;27(2):169–90. doi: 10.1016/s0145-2134(02)00541-0 12615092

[49] Yu J, Park J, Park D, Ryu S, Ha J. Validation of the Korean Childhood Trauma Questionnaire: the practical use in counselling and therapeutic intervention. Korean J Health Psychol. 2009;14(3):563–78.

[50] Cohen S, Kamarck T, Mermelstein R. A Global Measure of Perceived Stress. J Health Soc Behav. 1983;24(4):385. doi: 10.2307/2136404 6668417

[51] Lee J, Shin C, Ko YH, Lim J, Joe SH, Kim S, et al. The reliability and validity studies of the Korean version of the Perceived Stress Scale. Korean J Psychosom Med. 2012;20(2):127–34.

[52] Bagby RM, Parker JD, Taylor GJ. The twenty-item Toronto Alexithymia Scale--I. Item selection and cross-validation of the factor structure. J Psychosom Res. 1994;38(1):23–32. doi: 10.1016/0022-3999(94)90005-1 8126686

[53] Chung U-S, Rim H-D, Lee Y-H, Kim S-H. Comparison of reliability and validity of three Korean versions of the 20-item Toronto Alexithymia Scale. Korean J Psychosom Med. 2003;11(1):77–88.

[54] Preece D, Becerra R, Robinson K, Dandy J. Assessing Alexithymia: Psychometric Properties and Factorial Invariance of the 20-Item Toronto Alexithymia Scale in Nonclinical and Psychiatric Samples. J Psychopathol Behav Assess. 2017;40(2):276–87. doi: 10.1007/s10862-017-9634-6

[55] Reise SP, Bonifay WE, Haviland MG. Scoring and modeling psychological measures in the presence of multidimensionality. J Pers Assess. 2013;95(2):129–40. doi: 10.1080/00223891.2012.725437 23030794

[56] Chan J, Becerra R, Weinborn M, Preece D. Assessing Alexithymia across Asian and Western Cultures: Psychometric Properties of the Perth Alexithymia Questionnaire and Toronto Alexithymia Scale-20 in Singaporean and Australian Samples. J Pers Assess. 2023;105(3):396–412. doi: 10.1080/00223891.2022.2095641 35900047

[57] Baldwin JR, Wang B, Karwatowska L, Schoeler T, Tsaligopoulou A, Munafò MR, et al. Childhood Maltreatment and Mental Health Problems: A Systematic Review and Meta-Analysis of Quasi-Experimental Studies. Am J Psychiatry. 2023;180(2):117–26. doi: 10.1176/appi.ajp.20220174 36628513 PMC7614155

[58] Gardner MJ, Thomas HJ, Erskine HE. The association between five forms of child maltreatment and depressive and anxiety disorders: A systematic review and meta-analysis. Child Abuse Negl. 2019;96:104082. doi: 10.1016/j.chiabu.2019.104082 31374447

[59] Yue Y, Wang Y, Yang R, Zhu F, Yang X, Lu X, et al. Gender difference in the associations of childhood maltreatment and non-suicidal self-injury among adolescents with mood disorders. Front Psychiatry. 2023;14:1162450. doi: 10.3389/fpsyt.2023.1162450 37304441 PMC10248456

[60] Gratz KL, Chapman AL. The role of emotional responding and childhood maltreatment in the development and maintenance of deliberate self-harm among male undergraduates. Psychol Men Masc. 2007;8(1):1–14. doi: 10.1037/1524-9220.8.1.1

[61] Power J, Gobeil R, Beaudette JN, Ritchie MB, Brown SL, Smith HP. Childhood Abuse, Nonsuicidal Self-Injury, and Suicide Attempts: An Exploration of Gender Differences in Incarcerated Adults. Suicide Life Threat Behav. 2016;46(6):745–51. doi: 10.1111/sltb.12263 27291490

[62] Swannell S, Martin G, Page A, Hasking P, Hazell P, Taylor A, et al. Child maltreatment, subsequent non-suicidal self-injury and the mediating roles of dissociation, alexithymia and self-blame. Child Abuse Negl. 2012;36(7–8):572–84. doi: 10.1016/j.chiabu.2012.05.005 22858062

[63] Klonsky ED, Moyer A. Childhood sexual abuse and non-suicidal self-injury: meta-analysis. Br J Psychiatry. 2008;192(3):166–70. doi: 10.1192/bjp.bp.106.030650 18310572

[64] Brick L, Nugent N, Armey M. Affective variability and childhood abuse increase the risk for nonsuicidal self-injury following psychiatric hospitalization. J Trauma Stress. 2021;34(6):1118–31. doi: 10.1002/jts.22739 34655112

[65] Nock MK, Kessler RC. Prevalence of and risk factors for suicide attempts versus suicide gestures: analysis of the National Comorbidity Survey. J Abnorm Psychol. 2006;115(3):616–23. doi: 10.1037/0021-843X.115.3.616 16866602

[66] Di Pierro R, Sarno I, Perego S, Gallucci M, Madeddu F. Adolescent nonsuicidal self-injury: the effects of personality traits, family relationships and maltreatment on the presence and severity of behaviours. Eur Child Adolesc Psychiatry. 2012;21(9):511–20. doi: 10.1007/s00787-012-0289-2 22722662

[67] Andersson H, Aspeqvist E, Dahlström Ö, Svedin CG, Jonsson LS, Landberg Å, et al. Emotional Dysregulation and Trauma Symptoms Mediate the Relationship Between Childhood Abuse and Nonsuicidal Self-Injury in Adolescents. Front Psychiatry. 2022;13:897081. doi: 10.3389/fpsyt.2022.897081 35966492 PMC9366744

[68] Fan X, Liu Y, Liu Q, Zhao X, Zhou Y, Liu L. The impact of sexual abuse in childhood on adolescents’ non-suicidal self-injury behavior: A moderated mediation model. J Affect Disord. 2025;373:421–8. doi: 10.1016/j.jad.2024.12.111 39755129

[69] Yao J, Zou Y, Luo Q, Luo Y, Chunghon T, Shang H. Social Support and Depression Mediate the Relationship Between Childhood Trauma and Nonsuicidal Self-Injury. Clin Psychol Psychother. 2025;32(1):e70030. doi: 10.1002/cpp.70030 39868908

[70] Victor SE, Muehlenkamp JJ, Hayes NA, Lengel GJ, Styer DM, Washburn JJ. Characterizing gender differences in nonsuicidal self-injury: Evidence from a large clinical sample of adolescents and adults. Compr Psychiatry. 2018;82:53–60. doi: 10.1016/j.comppsych.2018.01.009 29407359 PMC5845831

[71] Glassman LH, Weierich MR, Hooley JM, Deliberto TL, Nock MK. Child maltreatment, non-suicidal self-injury, and the mediating role of self-criticism. Behav Res Ther. 2007;45(10):2483–90. doi: 10.1016/j.brat.2007.04.002 17531192 PMC2034449

[72] Burns EE, Jackson JL, Harding HG. Child Maltreatment, Emotion Regulation, and Posttraumatic Stress: The Impact of Emotional Abuse. J Aggress Maltreat Trauma. 2010;19(8):801–19. doi: 10.1080/10926771.2010.522947

[73] O’Mahen HA, Karl A, Moberly N, Fedock G. The association between childhood maltreatment and emotion regulation: two different mechanisms contributing to depression?. J Affect Disord. 2015;174:287–95. doi: 10.1016/j.jad.2014.11.028 25528000

[74] Jarvers I, Heidingsfelder E, Ecker A, Kandsperger S, Brunner R, Schleicher D. Characteristics of self-injurious behaviour and early traumatic experiences: associations with emotional reactivity, depression and aggression in university students. BJPsych Open. 2025;11(2):e45. doi: 10.1192/bjo.2024.862 40065550 PMC12001921

[75] Kiekens G, Bruffaerts R, Nock MK, Van de Ven M, Witteman C, Mortier P, et al. Non-suicidal self-injury among Dutch and Belgian adolescents: Personality, stress and coping. Eur Psychiatry. 2015;30(6):743–9. doi: 10.1016/j.eurpsy.2015.06.007 26260264

[76] Boyne H, Hamza CA. Depressive Symptoms, Perceived Stress, Self-Compassion and Nonsuicidal Self-Injury Among Emerging Adults: An Examination of the Between and Within-Person Associations Over Time. Emerg Adulthood. 2022;10(5):1269–85. doi: 10.1177/21676968211029768 36111318 PMC9465554

[77] Ammerman BA, Wilcox KT, O’Loughlin CM, McCloskey MS. Characterizing the choice to disclose nonsuicidal self-injury. J Clin Psychol. 2021;77(3):683–700. doi: 10.1002/jclp.23045 32869874

[78] Ewing L, Hamza CA, Willoughby T. Stressful Experiences, Emotion Dysregulation, and Nonsuicidal Self-Injury among University Students. J Youth Adolesc. 2019;48(7):1379–89. doi: 10.1007/s10964-019-01025-y 31025157

[79] Bedi R, Muller RT, Classen CC. Cumulative risk for deliberate self-harm among treatment-seeking women with histories of childhood abuse. Psychol Trauma. 2014;6(6):600–9. doi: 10.1037/a0033897

[80] Kick L, Schleicher D, Ecker A, Kandsperger S, Brunner R, Jarvers I. Alexithymia as a mediator between adverse childhood events and the development of psychopathology: a meta-analysis. Front Psychiatry. 2024;15:1412229. doi: 10.3389/fpsyt.2024.1412229 39011338 PMC11246998

[81] Claes L, Vandereycken W, Vertommen H. Self-injury in female versus male psychiatric patients: A comparison of characteristics, psychopathology and aggression regulation. Pers Individ Dif. 2007;42(4):611–21. doi: 10.1016/j.paid.2006.07.021

[82] Zhou J, Zhang J, Huang Y, Zhao J, Xiao Y, Zhang S, et al. Associations between coping styles, gender, their interaction and non-suicidal self-injury among middle school students in rural west China: A multicentre cross-sectional study. Front Psychiatry. 2022;13:861917. doi: 10.3389/fpsyt.2022.861917 36016979 PMC9395723

[83] Howe-Martin LS, Murrell AR, Guarnaccia CA. Repetitive nonsuicidal self-injury as experiential avoidance among a community sample of adolescents. J Clin Psychol. 2012;68(7):809–29. doi: 10.1002/jclp.21868 22589002

[84] Rasmussen S, Hawton K, Philpott-Morgan S, O’Connor RC. Why Do Adolescents Self-Harm?. Crisis. 2016;37(3):176–83. doi: 10.1027/0227-5910/a000369 26831210

[85] Moloney F, Amini J, Sinyor M, Schaffer A, Lanctôt K, Mitchell RHB. Research Review: Sex differences in the clinical correlates of nonsuicidal self-injury in adolescents - a systematic review. J Child Psychol Psychiatry. 2025;66(8):1263–73. doi: 10.1111/jcpp.14114 39825677 PMC12267678

[86] Steinhoff A, Ribeaud D, Kupferschmid S, Raible-Destan N, Quednow BB, Hepp U, et al. Self-injury from early adolescence to early adulthood: age-related course, recurrence, and services use in males and females from the community. Eur Child Adolesc Psychiatry. 2021;30(6):937–51. doi: 10.1007/s00787-020-01573-w 32572615 PMC8140957

[87] Danese A, Widom CS. The Subjective Experience of Childhood Maltreatment in Psychopathology. JAMA Psychiatry. 2021;78(12):1307–8. doi: 10.1001/jamapsychiatry.2021.2874 34643689

[88] Danese A, Widom CS. Objective and subjective experiences of child maltreatment and their relationships with psychopathology. Nat Hum Behav. 2020;4(8):811–8. doi: 10.1038/s41562-020-0880-3 32424258

[89] Diamond M. Sex and Gender are Different: Sexual Identity and Gender Identity are Different. Clin Child Psychol Psychiatry. 2002;7(3):320–34. doi: 10.1177/1359104502007003002

[90] Hartung CM, Lefler EK. Sex and gender in psychopathology: DSM-5 and beyond. Psychol Bull. 2019;145(4):390–409. doi: 10.1037/bul0000183 30640497

[91] Liu RT. Temporal Trends in the Prevalence of Nonsuicidal Self-injury Among Sexual Minority and Heterosexual Youth From 2005 Through 2017. JAMA Pediatr. 2019;173(8):790–1. doi: 10.1001/jamapediatrics.2019.1433 31157883 PMC6547150

[92] Rogers ML, Taliaferro LA. Self-Injurious Thoughts and Behaviors Among Sexual and Gender Minority Youth: a Systematic Review of Recent Research. Curr Sex Health Rep. 2020;12(4):335–50. doi: 10.1007/s11930-020-00295-z

[93] Li S, Zhang B, Guo Y, Zhang J. The association between alexithymia as assessed by the 20-item Toronto Alexithymia Scale and depression: A meta-analysis. Psychiatry Res. 2015;227(1):1–9. doi: 10.1016/j.psychres.2015.02.006 25769520

[94] Hendryx MS, Haviland MG, Shaw DG. Dimensions of alexithymia and their relationships to anxiety and depression. J Pers Assess. 1991;56(2):227–37. doi: 10.1207/s15327752jpa5602_4 2056418

[95] Preece DA, Petrova K, Mehta A, Sikka P, Gross JJ. Alexithymia or general psychological distress? Discriminant validity of the Toronto Alexithymia Scale and the Perth Alexithymia Questionnaire. J Affect Disord. 2024;352:140–5. doi: 10.1016/j.jad.2024.01.271 38320659

[96] Brausch AM, Muehlenkamp JJ. Perceived effectiveness of NSSI in achieving functions on severity and suicide risk. Psychiatry Res. 2018;265:144–50. doi: 10.1016/j.psychres.2018.04.038 29709788 PMC5984167

